# Coordinated Regulation of Metabolic Transporters and Migration/Invasion by Carbonic Anhydrase IX

**DOI:** 10.3390/metabo8010020

**Published:** 2018-03-08

**Authors:** Paul C. McDonald, Mridula Swayampakula, Shoukat Dedhar

**Affiliations:** 1 Department of Integrative Oncology, BC Cancer Research Centre, Vancouver, BC V5Z 1L3, Canada; pmcdonal@bccrc.ca (P.C.M.); mswayampak@bccrc.ca (M.S.); 2 Department of Biochemistry and Molecular Biology, University of British Columbia, Vancouver, BC V6T 1Z3, Canada

**Keywords:** hypoxia, carbonic anhydrase IX, cancer metabolism, transporter, integrin, MMP14, migration, invasion, metastasis

## Abstract

Hypoxia is a prominent feature of the tumor microenvironment (TME) and cancer cells must dynamically adapt their metabolism to survive in these conditions. A major consequence of metabolic rewiring by cancer cells in hypoxia is the accumulation of acidic metabolites, leading to the perturbation of intracellular pH (pHi) homeostasis and increased acidosis in the TME. To mitigate the potentially detrimental consequences of an increasingly hypoxic and acidic TME, cancer cells employ a network of enzymes and transporters to regulate pH, particularly the extracellular facing carbonic anhydrase IX (CAIX) and CAXII. In addition to the role that these CAs play in the regulation of pH, recent proteome-wide analyses have revealed the presence of a complex CAIX interactome in cancer cells with roles in metabolite transport, tumor cell migration and invasion. Here, we explore the potential contributions of these interactions to the metabolic landscape of tumor cells in hypoxia and discuss the role of CAIX as a hub for the coordinated regulation of metabolic, migratory and invasive processes by cancer cells. We also discuss recent work targeting CAIX activity using highly selective small molecule inhibitors and briefly discuss ongoing clinical trials involving SLC-0111, a lead candidate small molecule inhibitor of CAIX/CAXII.

## 1. Introduction

As tumors develop, cancer cells must reprogram their metabolism to meet the demands of energy production and biosynthesis. The availability of nutrients and the configuration of the cellular metabolic network collaborate to determine how cancer cells perform core metabolic functions, including energy production, biomass accumulation and control of the redox state [1]. The expanding knowledge base surrounding metabolism in cancer has resulted in the identification of several hallmarks of cancer metabolism, including, but not limited to, deregulated uptake of glucose and amino acids, use of opportunistic modes of nutrient acquisition and dynamic metabolic interactions with the tumor microenvironment (TME) [2].

A consequence of the proliferation of cancer cells beyond the reach of established blood vessels is the development of intratumoral hypoxia, defined as regions exhibiting low partial pressure of oxygen (O_2_) [3]. Hypoxia is a prominent feature of the TME and its presence results in the stabilization by cancer cells of hypoxia-inducible factor 1 alpha (HIF-1α), the master regulator of the hypoxic response, leading to the upregulation of a plethora of gene products geared toward the protection of tumor cells against hypoxic stress [4]. Biological responses of the tumor to hypoxia include the induction of angiogenesis, resulting in the formation of a dysfunctional vasculature that serves to perpetuate poor perfusion and exacerbate hypoxia, and dynamic adaptation of cancer cell metabolism to enable the acquisition and use of nutrients and metabolites from an increasingly nutrient-poor, low-O_2_ environment, thereby maintaining viability and enabling continued proliferation [2,5].

Metabolically, hypoxia reduces the amount of O_2_ available for oxidative phosphorylation and cancer cells respond to this challenge by shifting toward the use of glycolysis for respiration [1,2,3]. This shift is coupled with the use of alternative fuel sources, including glutamine and fatty acids, optimization of the efficiency of oxidative phosphorylation and use of the tricarboxylic acid (TCA) cycle to generate metabolic precursors [1,2]. A major consequence of metabolic rewiring by cancer cells in hypoxia is the increased production and accumulation of acidic metabolites, particularly lactate, carbon dioxide (CO_2_) and protons (H^+^) [5]. The development of acidosis in the hypoxic TME leads to the perturbation of intracellular pH (pHi) homeostasis, a situation which rapidly impinges on cellular viability and drives cancer cells to engage compensatory survival mechanisms.

To mitigate the potentially detrimental consequences of an increasingly hypoxic and acidic TME, cancer cells employ a network of enzymes and transporters that work in concert to provide effective pH regulation [5]. Critical components of this pH regulatory system are carbonic anhydrases (CAs), particularly extracellular-facing carbonic anhydrase IX (CAIX) and CAXII [6,7,8]. In particular, CAIX is a HIF-1α-induced, cell-surface enzyme that regulates pHi and promotes tumor cell survival [6,8]. In addition to hypoxia, which is a major driver of CAIX expression by cancer cells [6,9], CAIX can also be induced in normoxia by high cell-density-mediated pseudohypoxia [10,11], and by hypoxia-independent mechanisms such as lactate- [12] and redox-mediated [13] stabilization of HIF-1α. CAIX is widely regarded as a prominent biomarker of poor patient prognosis and treatment resistance for many solid cancers [9]. Several studies have now demonstrated the critical role of CAIX in the growth and metastasis of multiple types of cancers [14,15,16,17], and recent data have suggested an important role of CAIX in tumor cell migration [18,19] and invasion [17,20,21,22]. 

The multifaceted role of CAIX in cancer cell biology, coupled with the relative paucity of available data regarding physiologically-relevant associations between CAIX and putative interacting proteins in cancer cells, has driven the need for studies aimed at elucidating the components comprising the CAIX interactome. As part of this research focus, recent investigations have been undertaken to identify proximal CAIX-interacting proteins using an unbiased proteomic screen centered on proximity-dependent biotin identification (BioID) technology [22]. These studies have uncovered the presence of an intricate CAIX interactome in cancer cells that controls important functional parameters essential to the processes of pH regulation, transport of metabolic intermediates, cell migration and invasion [22]. In particular, the data have revealed the presence of two major classes of membrane proteins that are associated with CAIX, specifically metabolic transport proteins and cell adhesion/migration/invasion proteins. Here, we explore the potential contributions of these interactions to the metabolic landscape of tumor cells in hypoxia [23,24] and discuss the role of CAIX as a key hub for the coordinated regulation of metabolic, migratory and invasive processes by cancer cells. We also discuss recent work targeting CAIX activity in pre-clinical models using small molecule inhibitors of CAIX and briefly describe ongoing clinical trials using a lead compound, SLC-0111.

## 2. Membrane-Localized Metabolic Transport Proteins

**Figure 1:**
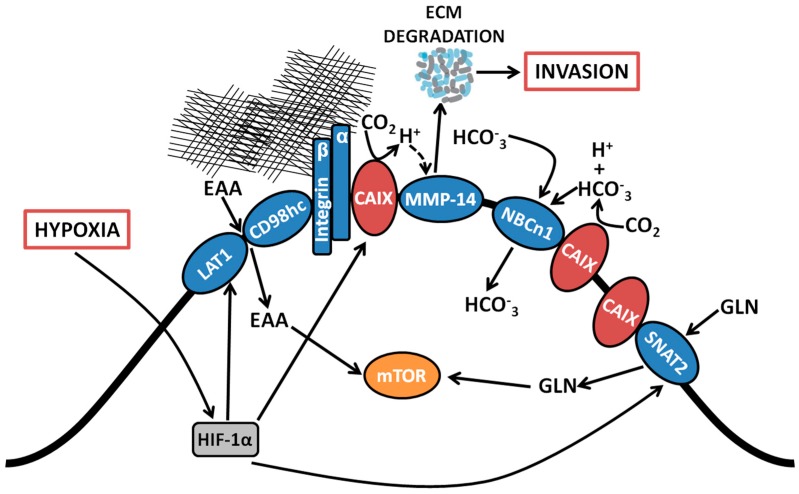
Coordinated regulation by CAIX of amino acid and bicarbonate transporters, and migration/invasion through interaction with integrins and MMP14. Proteomic analyses revealed associations between CAIX and several membrane-bound transport proteins. CAIX couples with bicarbonate transporters to facilitate influx of HCO_3_^−^. CAIX associates with amino acid transporters important for the import of both essential amino acids and the conditionally essential amino acid glutamine, which serve as alternative metabolic fuels and biosynthetic precursors for use by cancer cells. CAIX also forms novel associations with collagen- and laminin-binding integrins localized at pseudopodia-like protrusions at the leading edge of migrating cells. Finally, CAIX potentiates MMP14 activity at invadopodia through donation of H^+^ released by CAIX-mediated CO_2_ hydration. EAA, essential amino acids; LAT1, large neutral amino acid transporter 1; CD98hc, cluster of differentiation 98 heavy chain; CAIX, carbonic anhydrase IX, NBCn1, sodium-dependent electroneutral bicarbonate transporter n1; SNAT2, sodium-coupled neutral amino acid transporter 2; MMP14, matrix metallopeptidase 14; GLN, glutamine; HCO_3_^−^, bicarbonate; CO_2_, carbon dioxide; H^+^, proton; ECM, extracellular matrix.

Among the CAIX-associating proteins identified using the BioID platform, associations with membrane-localized metabolic transporters from several functional classes, including bicarbonate (HCO_3_^−^) transporters and amino acid (AA) transporters were observed [22]. Specifically, CAIX is proximally associated with the sodium-dependent electroneutral bicarbonate transporter n1 (NBCn1), encoded by the gene solute-like carrier (SLC) 4A7 (*SLC4A7*). CAIX also associates with a suite of proteins involved in AA transport, including the L-type AA transporter, LAT1 (*SLC7A5*), the AA transport heavy chain subunit, CD98hc (*SLC3A2*) and the glutamine transporters alanine-serine-cysteine-preferring transporter 2 (ASCT2; *SLC1A5*) and sodium-coupled neutral amino acid transporter 2 (SNAT2; *SLC38A2*). The interaction of CAIX with this diverse array of membrane-localized metabolic transport proteins suggests that it may serve as a central regulator of metabolic processes by cancer cells during hypoxic stress (Figure 1). Each of these interactions is discussed further below.

### 2.1. Bicarbonate Transporters

A key functional parameter of pHi regulation is the efficient, effective capture and import of HCO_3_^−^ produced by CAIX-mediated hydration of CO_2_ at the extracellular surface to buffer intracellular acidosis. CAIX has previously been proposed to associate with Na^+^/HCO_3_^−^ co-transporters to form a transport “metabolon”, defined as a protein complex composed of metabolic enzymes that function to optimize the transfer of metabolic intermediates between active sites, bypassing the need for equilibration with bulk buffer [5]. While functional coordination of enzymes in metabolons has been reported [24], the existence of a proximal or physical association between the enzymes in metabolons generally and between CAIX and HCO_3_^−^ transporters, specifically, has been controversial [5]. Studies have suggested that the expression of various HCO_3_^−^ transporters is upregulated in hypoxia in a complex, cell-type-dependent fashion [25,26]. Furthermore, hypoxia-induced expression of the HCO_3_^−^ transporter *SLC4A9* has been identified as having an essential role in tumor progression [26], while constitutive expression of electrogenic Na^+^/HCO_3_^−^ co-transporter (*SLC4A4*) has been reported to play a role in breast and colon cancer cell proliferation, migration and pHi regulation [25]. However, these studies did not directly investigate the presence of an association between CAIX and the HCO_3_^−^ transporters.

Interrogation of the components of the CAIX interactome in triple negative breast cancer cells by employing unbiased proteome-wide strategies such as BioID has now affirmed the presence of a proximal association of CAIX with the electroneutral bicarbonate transporter NBCn1 (*SLC4A7*) (Figure 1) [22]. The NBCn1 transporter is implicated in breast cancer susceptibility [27] and knockout of NBCn1 in a mouse model of breast cancer has been reported to increase the latency of tumor development and impair tumor growth [28], indicating the importance of this transporter in breast cancer and alluding to the pathophysiological relevance of an interactive metabolon involving CAIX and HCO_3_^−^ transporters. The proximal association of CAIX with NBCn1 is congruent with its participation in the setting of a metabolon and indicates that CAIX induced by hypoxia may couple with HCO_3_^−^ transporters already present on the cell surface. While the precise molecular mechanisms remain to be determined, the presence of the CAIX-NBCn1 association demonstrates that CAIX can couple with specific HCO_3_^−^ transporters, potentially facilitating the local production of HCO_3_^−^ for subsequent capture and transfer into the cell to efficiently regulate pHi.

### 2.2. Essential Amino Acid Transporters

In addition to associating with proteins that rely directly on the catalytic function of CAIX, proteome-wide analyses have uncovered novel associations between CAIX and components of the amino acid (AA) transport system (Figure 1), including the large neutral amino acid transporter 1 (LAT1) and CD98 heavy chain (CD98hc), which themselves form a heterodimeric complex and function as a transporter of essential AAs (EAAs) that cannot be produced de novo by mammalian cells, such as leucine (leu), and the glutamine transporters ASCT2 and SNAT2 (discussed in Section 2.3 below) [22]. Such interactions raise the exciting possibility that cancer cells which have undergone metabolic reprogramming and require augmented capacity for nutrient acquisition to support cell growth in hypoxia may recruit CAIX to assist in the coordinated regulation of AA transport. While the molecular mechanisms and functional contribution of coupling CAIX to AA transporters remains an area for future investigation, it is clear that, in addition to skewing their metabolism toward glucose utilization, cancer cells rely on additional fuels to carry out core metabolic functions, including energy production and biosynthetic processes [1]. The presence of hypoxia further limits nutrient acquisition from the TME [3], making the capacity to acquire and utilize alternative fuels and nutrients particularly advantageous. Furthermore, overexpression of LAT1 is a negative prognostic indicator for many cancers [29] and LAT1 activity was found to be required for tumor growth in conditions of hypoxia and nutrient depletion [30], similar to conditions that induce CAIX expression.

It is notable that LAT1 has been shown to promote the activity of mTORC1, a master regulator of cell growth and metabolism [30], and sustained activation of mTORC1 requires the presence of intracellular leucine (leu), an EAA imported by LAT1 [29]. Recently published studies have shown that pharmacologic inhibition of CAIX activity in vivo in a model of glioblastoma multiforme (GBM)—when used in combination with the standard of cancer chemotherapy, temozolomide—results in an altered flux of AAs, including essential AAs such as leucine [31], potentially linking CAIX to EAA transport through an association with LAT1, although the mechanism remains to be determined. These findings suggest that CAIX may play a role in regulating metabolic functions in cancer cells beyond pH homeostasis and point to the potential involvement of CAIX in coordinating the regulation of EAA transport by cancer cells in hypoxia.

### 2.3. Glutamine Transporters

While metabolic reprogramming by cancer cells clearly results in a shift toward the use of glucose as fuel source, it is now recognized that tumor cells are capable of using diverse array of nutrients, in particular glutamine, to support metabolic and biosynthetic functions. The increased use of glutamine by cancer cells as an alternative fuel source, combined with nutrient delivery inadequacies brought on by the deregulated tumor vascular supply, results in the selective depletion of glutamine from the TME [32]. Under these conditions, glutamine is considered to be a conditionally essential AA and cancer cells must find ways to augment glutamine acquisition and uptake. For example, cancer cells may upregulate ASCT2, the major transporter for glutamine uptake [32,33]. Furthermore, cancer cells may upregulate other sodium neutral amino acid transporters, including SNAT1 and SNAT2, as a way of supplementing uptake by ASCT2 [33,34].

Metabolically, glutaminolysis contributes to the production of intracellular CO_2_ via decarboxylation of metabolic intermediates [5]. Thus, association of CAIX with glutamine transporters such as ASCT2 and SNAT2 potentially couples glutamine import and metabolism with the effective management of CO_2_ production (Figure 1). Furthermore, the association of CAIX with the glutamine transporter, SNAT2, an interaction specifically identified by the BioID analysis [22], together with its association with the essential AA transporter LAT1, suggests an active role for CAIX in coordinating the regulation of AA flux in general, especially since the import of EAA such as leu is coupled with glutamine efflux [2]. Thus, it is tempting to speculate that CAIX contributes functionally to cell energetics and biosynthesis in the context of hypoxia.

## 3. Cell Adhesion/Migration/Invasion Proteins

A growing body of evidence supports a role of CAIX as a key regulator of cancer cell migration, invasion and metastasis. For example, studies have demonstrated that the genetic depletion of CAIX reduces breast cancer invasion and metastasis [15,17] and pharmacologic inhibition of CAIX activity serves to inhibit metastasis in pre-clinical models of cancer [15,17,35]. Furthermore, recent analyses have shown that CAIX is a critical functional mediator of invasion in vitro in the biologically-relevant context of hypoxia [22]. Indeed, recent investigations have shown that CAIX is requisite for the invasion of tumor cells through matrices, including matrigel and type 1 collagen. Furthermore, it is probable that multiple regions of the CAIX protein, including the proteoglycan-(PG)-like domain and intracellular domain, contribute functionally to invasion, and that the catalytic activity of CAIX is necessary for the invasive process, given that invasion is abrogated in the presence of a small molecule inhibitor of CAIX [22].

In support of a role of CAIX in migration and invasion, proteomic and co-immunoprecipitation analyses have revealed novel associations of CAIX with several proteins involved in cell adhesion, matrix remodeling and invasion. In particular, CAIX associates with a compendium of integrin subunits, specifically integrin β1 (ITGB1), integrin α2 (ITGA2), integrin α3 (ITGA3), integrin α5 (ITGA5) and integrin α6 (ITGA6) [22], highlighting a potential role of CAIX in coordinating the regulation of collagen- and laminin-binding integrins to control cancer cell adhesion, a critical process involved in migration and invasion (Figure 1). CAIX also associates with MMP14, a key player in the matrix degradation process required for successful invasion by cancer cells (Figure 1). The association of CAIX with these well-recognized effectors of migration and invasion suggests that these interactions may be functionally relevant in the formation and/or activity of protrusive invasive structures such as pseudopodia [36] and invadopodia [37] in hypoxia. The association of CAIX with integrins and MMP14 within these protrusive structures, together with potential mechanistic consequences of the interaction with MMP14, is discussed further below.

### 3.1. Association with Integrins and MMP14 in Pseudopodia

**Figure 2:**
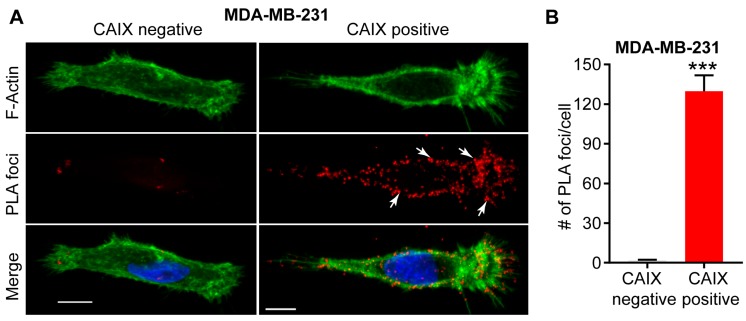
Interaction between CAIX and MMP14 as observed by proximity ligation assay (PLA). (**A**) Immunofluorence images showing the interaction of CAIX and MMP14 by PLA (red foci; arrows) in MDA-MB-231 cells depleted of CAIX using CRISPR-Cas technology (CAIX negative) or similar cells constitutively expressing CAIX (CAIX positive). Actin (green) and nuclei (blue) are shown for purposes of orientation. PLA-positive signals are concentrated in pseudopodia-like protrusions at the leading edge of migrating, CAIX-positive cells. Scale bar = 10 μm; (**B**) Quantification of PLA-positive foci. Data show the mean ± sem of *n* = 74 cells and are representative of 2 independent experiments. *** *p* < 0.001.

Examination of the membrane extensions formed by breast cancer cells cultured on collagen in hypoxia has demonstrated spatial localization of CAIX in association with integrins ITGB1 and ITGA2 in actin- and cofilin-positive, pseudopodia-like protrusions resembling lamellipodia (Figure 1) [22]. The association of CAIX with integrins was very evident at the leading edges of cells with a migratory phenotype, but was distinctly absent from focal adhesions, suggesting that CAIX associates with these proteins specifically in cellular regions involved in migration [22]. In addition to integrins, immunofluorescence analyses have shown that CAIX associates with MMP14, a matrix metalloprotease that counts collagen type I among its substrates, at pseudopodia-like protrusions resembling lamellipodia, suggesting that CAIX may function to regulate MMP14-mediated matrix degradation at these structures (Figure 1) [22]. Further analysis using a proximity ligation assay (PLA) has confirmed these results, demonstrating that CAIX and MMP14 reside in close proximity to one another in breast cancer cells (Figure 2).

It is interesting that CAIX associates both with integrins and, as discussed above, with CD98hc. While CD98hc is a component of the AA transport system, evidence also suggests that it plays a role in regulating integrin-mediated tissue stiffness [38]. Given these findings, it is possible that CAIX may provide a link between AA transport and integrin-mediated adhesion at membrane protrusions. The potential functional relevance of the interactions between CAIX, integrins and CD98hc remain to be elucidated by future research.

### 3.2. Functional Role of a CAIX-MMP14 Interaction at Invadopodia

In addition to its presence in pseudopodia-like protrusions, MMP14 is a well-established component of invadopodia, the protrusive, matrix degrading structures on the ventral surface of cells that concentrate and release proteases to enable ECM degradation, thereby facilitating invasion and metastasis by cancer cells [37,39,40]. Congruent with the findings that MMP14 is a component of the CAIX interactome in cancer cells and that CAIX localizes with MMP14 in pseudopodia-like protrusions, immunoflurescence analyses have now shown that, in breast cancer cells, CAIX specifically co-localizes with MMP14 at functional invadopodia, where it functions to regulate collagen degradation [22]. Furthermore, detailed biochemical examination of this interaction has revealed that the intracellular domain of CAIX interacts with MMP14 and that the interaction depends on putative phosphorylation sites positioned within the intracellular domain of CAIX [22]. Mechanistically, CAIX enhances MMP14-mediated collagen degradation by providing a local reservoir of H^+^ required for MMP14 catalytic activity [22] (Figure 1). Importantly, this novel mechanism for the regulation of MMP14-mediated invasion by CAIX is highly biologically relevant, given that extracellular acidosis is thought to activate proteases [41] and it has been reported that collagen degradation by MMP14 is increased in acidic pH [42]. Furthermore, the contribution of CAIX to the regulation of MMP14 activity at invadopodia is of particular importance in hypoxia. It is now understood that the pH regulatory protein Na^+^/H^+^ exchanger 1 (NHE1) is recruited to invadopodia, where it regulates invadopodia function by modulating pHi [43,44] and drives cofilin-dependent actin polymerization and recruitment of MMPs, including MMP14 [45]. As a consequence of its activity, NHE1 extrudes H^+^ into the extracellular environment, thereby contributing to extracellular acidosis. In regions of hypoxia, however, NHE1 activity is reduced [46] and NHE1 gene expression is reported to be low in basal type and triple negative breast cancers [47], whereas CAIX is expressed in over 50% of patients with this breast cancer subtype [15], a patient subset that also expresses MMP-14. Thus, the MMP14-CAIX interaction at invadopodia provides a putative mechanism for potentiation of MMP14 degradative activity in situations where the activity of NHE1 may be compromised.

It is also interesting that while the presence of CAIX at protrusive structures at the leading edge of cancer cells allude to a possible functional contribution by CAIX to the process of cancer cell migration; the interaction between CAIX and MMP14 at invadopodia suggests a scenario whereby CAIX actively modulates invasion via mechanisms that are independent of migration and that involve localized stimulation of MMP14 activity to regulate the degradation of collagen [22]. Recent studies have reported the presence and/or upregulation of CAIX at the invasive front of carcinomas in patients [15,48]. Similarly, MMP14 expression is associated with tumor progression, invasion and metastasis, and poor prognosis [49,50,51,52]. Taken together, these data suggest that the localization and association of CAIX and MMP14 may lead to MMP14 activation at invadopodia, providing a novel mechanism of invasion that can be incorporated into the arsenal of functional processes used by cancer cells for invasion and metastasis. 

## 4. Targeting CAIX Activity in Hypoxic Solid Tumors

Substantive research efforts in recent years have focused on the development, pre-clinical and clinical evaluation of therapeutic strategies targeting CAIX (and CAXII) in solid malignancies. The HIF-1-mediated, tumor-specific upregulation of CAIX, its localization at the cell surface, its highly restricted expression in normal tissues, the association of CAIX upregulation with poor prognosis and its functional relevance to tumor biology all serve as key properties for its use as a therapeutic target. To date, several studies have provided validation of targeting CAIX in multiple tumor models [14,15,16,17] and an array of potential therapeutic strategies to target CAIX have been developed, including the use of small molecule inhibitors of CAIX/CAXII activity [6,7].

Among a large number of CAIX/CAXII inhibitor compounds reported to date, the ureido-substituted benzenesulfonamides has been evaluated extensively for anti-tumor activity [15,17,31,53]. Pre-clinical studies in models of breast cancer have demonstrated the efficacy of the lead candidate CAIX/CAXII inhibitor, SLC-0111, in reducing tumor growth and inhibiting the formation of metastases [15,17]. Furthermore, administration of SLC-0111 in combination with conventional chemotherapy agents such as paclitaxel further inhibited tumor growth, compared to individual treatments [17]. Moving beyond breast cancer, recently published data demonstrate that targeting CAIX/CAXII is a promising therapeutic avenue in glioblastoma multiforme (GBM), when used in combination with standard of care chemotherapy. The Cancer Genome Atlas (TCGA) data from patients with GBM showed that high CAIX correlated with markedly reduced survival [31]. Treatment of a model of recurrent GBM with SLC-0111 in combination with temozolomide significantly delayed tumor growth, while treatment of an orthotopic patient derived xenograft (PDX) model of GBM with the combination resulted in increased survival [31]. Current studies using SLC-0111 in combination with gemcitabine in models of pancreatic cancer are yielding congruent data. Taken together, these findings indicate the potential for the broad use of CAIX/CAXII inhibitors in combination with standard of care chemotherapy to enhance therapeutic response, reduce toxicity, and combat therapeutic resistance across multiple cancer types [9]. In fact, SLC-0111 has now completed a multi-centre Phase I clinical trial (NCT02215850) in cancer patients. While the results of this first human trial are still to be published, SLC-0111 was shown to have a favorable safety profile and is now the subject of a Phase Ib trial targeting patients with CAIX-positive, metastatic pancreatic cancer.

## 5. Conclusions

In addition to the well-recognized role of CAIX in pH regulation, global proteomic initiatives have uncovered novel interactions of CAIX involved in metabolite transport and tumor cell migration and invasion, and new functional roles in regulating hypoxia-induced cancer cell invasion. In particular, CAIX associates with two protein classes, membrane-bound metabolic transport proteins and cell adhesion/migration/invasion proteins. The association of CAIX with metabolic transporters, especially with those that transport EAAs and glutamine, suggests an increasingly complex role of CAIX in coordinated regulation of cancer cell metabolism in hypoxia. Moreover, associations with integrins and MMP14 indicate novel roles of CAIX in modulating motility and invasion. The multifunctional capacity driven by the complexity of the CAIX interactome suggests that targeting its activity will exact substantive therapeutic benefits by interfering with several aspects of cancer biology, including metabolism, pH regulation, invasion and metastasis.
